# Protective effects of HSP Inducer on diazinon-exposed stellate sturgeon (*Acipenser stellatus*) fry: Insights on HSP70 gene expression, immune response, and enzyme indices

**DOI:** 10.1371/journal.pone.0294188

**Published:** 2023-11-13

**Authors:** Leila Vahdatiraad, Behrooz Heidari, Sevda Zarei, Tooraj Sohrabi, Hossein Ghafouri

**Affiliations:** 1 Faculty of Science, Department of Biology, University of Guilan, Rasht, Iran; 2 Department of Marine Sciences, The Caspian Sea Basin Research Center, University of Guilan, Rasht, Iran; 3 International Caspian Sturgeon Research Institute, Agricultural Research, Education and Extension Organization (AREEO), Rasht, Iran; ICAR Indian Institute of Agricultural Biotechnology, INDIA

## Abstract

Aquatic environments face frequent exposure to organophosphate pesticides, such as diazinon, which are frequently utilized in agriculture. The goal of this study was to evaluate the effects of diazinon exposure on fish and to investigate the potential of the HSP inducer (HSPi) in developing a defense mechanism. To achieve this, several factors were analyzed, including the HSP70 gene expression, levels of immunity markers (lysozyme, IgM, and C3), antioxidant status, and the activity of acetylcholine esterase (AChE). Stellate sturgeon (*Acipenser stellatus*) fry, was exposed to diazinon (25, 50, and 75% of 96h-LC_50_) for 6 days after pre-treatment with an HSP inducer (HSPi), TEX-OE^®^ (a prickly pear cactus extract), for 4 hours. Two HSPi concentrations, 100 and 200 mg.L^-1^, were used. Pre-treatment with HSPi significantly enhanced HSP70 gene expression in the gill and liver, as well as immune markers in the blood of *Acipenser stellatus*. Diazinon-treated groups exhibited higher antioxidant activities of SOD, CAT, and T-AOC. Increased activity also observed in control fish pre-treated with HSPi. However, stellate sturgeon receiving both diazinon and HSPi+diazinon experienced a significant decrease in AChE activity in comparison with control group. Cortisol levels were elevated in the fish that were subjected to diazinon. Those subjected to diazinon after receiving HSPi showed a significant decrease in cortisol levels. In conclusion, the study suggests that HSPi-mediated HSP70 induction may have a protective effect. The presence of an HSP inducer offers a potential strategy to mitigate the consequences of diazinon exposure in stellate sturgeon.

## 1. Introduction

Nowadays, herbal extracts have emerged as safe, effective, and cost-effective alternatives to conventional drugs, chemicals, and antibiotics in the treatment of diseases and fish immunity enhancement [1]. *Opuntia ficus indica*, commonly called as the prickly pear or Nopal cactus, is a plant that is abundant in polyphenols, vitamins, polyunsaturated fatty acids, and amino acids. These components have antioxidant properties and can help reduce inflammation [2]. In recent years, there has been a growing body of research and industry reports that present substantiating evidence for the diverse health and medicinal advantages associated with this particular cactus [3–7]. TEX-OE^®^, a derivative obtained from Nopal cactus, has received approval to be used in fish and shellfish aquaculture due to its ability to naturally induce high amounts of endogenous or host-derived heat shock proteins (HSPs) [8,9].

Among the HSP family, HSP70 holds significant biological importance in fish. This includes its role in stress resilience, immunological activities, and the maintenance of protein integrity [8,10–13]. Many research studies indicate that being subjected to different stress factors, such as extreme temperatures, pollutants, anoxia, parasitism, predation, or competition, can cause a temporary rise in the expression of HSP70. This upregulation of HSP70 serves as a protective mechanism for organisms to prevent potential cellular harm [14–17]. As a result, HSP70 is commonly used as a stress biomarker. It provides valuable insights into the correlation between stress and biological reactions in fish.

Diazinon is categorized as a moderate persistence organophosphate pesticide (OP). It poses a threat to non-target aquatic organisms, including fish, as it can contaminate groundwater through drainage and surface runoff from cultivated lands [18–20]. In the northern provinces of Iran, situated along the southern coast of the Caspian Sea, diazinon is extensively used. It accounts for up to 60% of the total pesticide usage in the country [21]. The concentrations of diazinon have been measured in various rivers entering the Caspian Sea. For example, in Golestan province, the Qara-Su showed concentrations ranging from 18.6 to 22.4 mg.L^-1^, while the Gorgan-rud exhibited concentrations of 6.74 to 6.89 mg.L^-1^ [22]. Additionally, in Tonkabon city, the drainage of rice fields yielded concentrations of 93.1 to 0.98 mg.L^-1^ [23,24]. Moreover, the distribution and accumulation of diazinon in three important fish species from five estuaries along the Caspian Sea ranged from 0.01 to 0.15 mg.Kg^-1^ [25]. The presence of diazinon and its metabolites in fish can lead to various effects, including oxidative stress [26,27], alterations in hematological parameters [28], endocrine imbalances [18], and hyperactivity [29]. Numerous studies [30–33] have demonstrated that diazinon possesses the capacity to elicit profound transformations in liver enzymes and the corresponding biochemical indices. Consequently, the evaluation of fish health following exposure to acute or chronic diazinon toxicity can be performed by determining the activity of antioxidant enzymes, including superoxide dismutase (SOD), catalase (CAT), total antioxidant (T-AOC), cortisol, as well as immune parameters including lysozyme, IgM, and complement component C3. Moreover, the inhibition of esterase activity, which results from the inhibition of acetylcholinesterase (AChE) activity by diazinon and its metabolites [34,35], represents a distinctive biomarker for fish exposed to diazinon, leading to disruptions in feeding, escape, and reproductive behaviors [36–38].

The stellate sturgeon (*Acipenser stellatus*) is one of five anadromous sturgeon species found in the Caspian Sea and relies on nearby rivers as significant natural spawning grounds [39,40]. Unfortunately, due to overfishing, habitat loss caused by river fragmentation, dam construction, and pollution, this species is critically endangered according to the latest assessment by the IUCN Red List [41]. To rehabilitate sturgeon populations, fish fry are released into rivers that flow into the Caspian Sea. In 2018, the Shahid Beheshti Sturgeon Restoration and Genetic Conservation Center (Rasht, Iran), released over 1.5 million sturgeon fry into these rivers [42]. However, post-release, there have been reports of considerable deaths and financial damages caused by environmental stresses [43,44]. Therefore, it is crucial to assess the fry’s ability to tolerate different stressors for a successful release into rivers.

This study focuses on exposing stellate sturgeon fry to an HSP inducer called TEX-OE^®^ and sublethal concentrations (25%, 50%, and 75% of the LC_50_) of diazinon. The study assesses HSP70 gene expression levels in the liver and gills, measuring antioxidant activity (SOD, CAT, and T-AOC) in the liver. It also examines immunity parameters and cortisol levels in the blood, and evaluates the enzyme activity of AChE in the brain tissue.

## 2. Materials and methods

### 2.1. Fish supply

Sturgeon fry weighing approximately 12.5 ± 2.5 g were provided from the Shahid Beheshti Sturgeon Breeding and Rearing Center in Guilan, Iran. It was transported to the Marine Biology laboratory at the University of Guilan. Prior to the experiment, the fish were provided with commercial sustenance (BioMar, France) at a dosage equivalent to 2% of their bodily mass twice a day. The fish were acclimated to a well-aerated fiberglass tank with a capacity of 2000 L for two weeks, with approximately 20% of tank water changed daily. Temperature (18 ± 1°C), dissolved oxygen (~8–9 mg.L^-1^), and pH (~7–8) were kept constant throughout the experiment. No mortality was observed among the specimens throughout the acclimation period.

### 2.2. Preparation of HSP inducer

To induce HSP expression, the fish fry were exposed to TEX-OE^®^ Nopal endurance capsules (Source Naturals, INC, Box 2118, Santa Cruz, CA 95062) at two concentrations: 100 and 200 mg.L^-1^ [3,5,9]. The capsules were dissolved in sterilized distilled water to prepare the HSP inducer. The sturgeon fry were exposed to a duration of 4 hours in the TEX-OE^®^ solution, following the procedural technique outlined by [45]. Following exposure, the fish were returned to the experimental tank and then treated with diazinon.

### 2.3. Experimental design

According to [46], the 96-hour LC50 of diazinon for stellate sturgeons was estimated to be 2.54 mg.L^-1^. In the experiment, fish fry were exposed to diazinon levels of 25% (D_25_), 50% (D_50_), and 75% (D_75_) of the diazinon LC_50_. A total of 360 fish were used in the study. Each treatment consisted of three repetitions and there were 10 fish in each aquarium. The treated groups were as follows:

1. Control group (Control): Fish that were not exposed to HSPi or diazinon.
2. Fish that received 100 and 200 mg.L^-1^ of HSPi: H_100_ and H_200_.
3. Fish receiving sublethal diazinon doses: D_25_, D_50_, and D_75_.
4. Fish that received a combination of both HSPi and diazinon (first HSPi and then diazinon): H_100_+D_25_, H_100_+D_50_, H_100_+D_75_, H_200_+D_25_, H_200_+D_50_, H_200_+D_75_.

The temperature was around 18 ± 1°C, dissolved oxygen levels were kept between 8–9 mg.L^-1^, and the pH was maintained around 7–8. Throughout the experimental period, the 40 L aquariums were aerated to ensure sufficient oxygen supply, but no water changes were made.

### 2.4. Blood and tissue sampling

9 fish were sampled from each group and time point after being exposed to diazinon for 1, 3, and 6 days. The fish were anesthetized with clove oil, and their blood was taken from the caudal vein via insulin syringes. After centrifugation at 3000 rpm for 5 minutes, the serum was stored at -80°C for subsequent analysis of immune parameters, including lysozyme, IgM, C3, and cortisol concentrations. The fish were euthanized with an overdose of the anesthetic. The gills and liver tissues were quickly frozen using liquid nitrogen and kept at a temperature of -80°C until the HSP70 gene expression analysis. The liver and brain tissues were manually homogenized, weighed, and placed in 1.0 mL of Triton X-100 buffer to measure antioxidant enzymes and AChE activity. The mixture was held on ice for 10 minutes. After centrifugation at 4°C, 15 min at 12,000 rpm, the supernatant was kept at -80°C until further evaluation.

Approval for the experimental methods utilized was given by the National Committee for Ethics in Biochemical Research, located in Tehran and functioning under Iran’s Ministry of Health and Medical Education (Reference Number 61530).

### 2.5. HSP70 gene expression

To extract total RNA, RNX-plus reagent (SINACLON, Iran) was used following the manufacturer’s instructions. In order to remove DNA contamination from total RNA before starting the cDNA synthesis, Rnase-free Dnase was used (After diluting extracted RNA, 1 μL enzyme, 1 μL buffer, and in the next step 1 μL of EDTA was added) (SINACLON, Iran). The RNA’s quality was evaluated by using electrophoresis on a 1.2% Agarose gel. The amount of RNA was measured using a NanoDrop spectrophotometer (NanoDrop one C, USA). The first strand of cDNA (SINACLON kit, Iran) was used for cDNA synthesis. The steps were performed according to the kit protocol. The total volume required for a cDNA synthesis reaction was 12 μL. In addition, the expression levels were standardized using the GAPDH gene as a reference gene. The real-time qRT-PCR primers for the HSP70 [47] and GAPDH [48] genes are shown in Table 1. To determine HSP70 gene expression in a 25 μL reaction volume, a Roche qPCR thermocycler (Roche light cycler 96 machines, Germany) was used. Each qRT-PCR reaction was conducted in triplicate with denaturation as a first step of 15 min at 95°C, followed by 40 cycles of amplification, 30 seconds at 63°C for primer annealing, and 30 seconds at 72°C for extension. The determination of quantification cycle (Ct) values was conducted by employing the light Cycler 961.1 application software (Roche, Germany). The relative changes in expression levels of the HSP70 and GAPDH gene were assessed using the 2^-(ΔΔCt)^ technique.

**Table 1:** Specific primers used for real-time quantitative polymerase chain reaction analysis of HSP70 and GAPDH.

Gene	Primers sequences	Product size (bp)
HSP70	forward: CGCTGGCCTTAATGTTCTCC	249
	reverse: GCGCTTGAACTCTGCAATGA	249
GAPDH	forward: ACACCCGCTCATCAATCTTT	114
	reverse: AGGTCCACGACTCTGTTGCT	114

### 2.6. Antioxidant enzymes

#### 2.6.1. Superoxide Dismutase (SOD)

SOD activity was determined according to Bolann and Ulvik’s method [49], using a commercial kit (ZellBio GmbH (Germany) (CAT No. ZB-SOD-96A). All steps were followed according to the manufacturer’s protocol. Under enzymatic conditions, the superoxide anion was transformed into oxygen and hydrogen peroxide in this kit. At the end of the experiment, a colorimetric measurement at 420 nm was obtained for the chromogen.

#### 2.6.2. Catalase (CAT)

In accordance with Goth’s method [50], CAT activity was identified by a commercial kit (ZellBio GmbH, Germany) (CAT No. ZB- CAT-96A). Next, this activity was determined calorimetrically at 405 nm.

#### 2.6.3. Total Antioxidant Activity (T-AOC)

T-AOC was measured by [51]. The T-AOC level was estimated using an ELISA kit (ZellBio GmbH, Germany) (CAT NO. ZB- TAC -96A) at 490 nm.

### 2.7. Immune parameters

#### 2.7.1. Lysozyme

Lysozyme activity was determined by a turbidimetric method [17], using a bacterium (*Micrococcus luteus*) lysis ability. To perform the assay, 25 μL of serum and 175 μL of *M*. *luteus* (Sigma-Aldrich, St. Louis, USA) with concentration of 0.2 mg.L^-1^ were added to 0.5 M phosphate-buffered saline (PBS), pH 6.2, in triplicate wells of a 96-well plate, with PBS used as a negative control. The optical density (OD) was recorded at 530 nm after 1 hour and 5 hours at 22°C. In this study, a 0.001 U.min^-1^ decrease in absorbance was considered equivalent to one unit of lysozyme activity.

#### 2.7.2 Complement Component C3

The C3 assay was measured using an ELISA sandwich (enzyme-linked immunosorbent assay) with an ELISA fish kit (Hangzhou East Biopharm Co., Ltd) [5]. The supernatant consisted of a monoclonal antibody coated with a monoclonal C3 fish antibody. An immune complex was created by incubating the plate at 37°C. Next, the biotin-labeled C3 antibody was added and blended with streptavidin-horseradish peroxidase. The unmixed enzyme was removed by washing the plate. After adding sulfuric acid, the liquid turned yellow. The OD value was measured by a microplate reader at 450 nm and converted to C3 concentrations using calibration.

#### 2.7.3. IgM

A similar method was employed to measure the IgM levels. The IgM level in the supernatant was determined using a monoclonal antibody by an ELISA quantification kit (Hangzhou Eastbiopharm Co., Ltd) [52]. An ELISA reader was used to measure OD at 450 nm. The measurements were done in triplicates.

### 2.8. Cortisol

The levels of serum cortisol were assessed using commercial ELISA kits (Nanjing Jincheng Institute, Nanjing, China). Color changes (450 nm) were spectrophotometrically evaluated according to the kit instructions [53].

### 2.9. Acetylcholinesterase (AchE)

AchE activity was evaluated based on [54] method. Brain tissue was weighted and suspended in 10 mL of sodium phosphate 0.1 M (Triton X-100) with pH 7.5. After homogenization on ice, the samples were centrifuged at 12,000 rpm for 15 min at 4°C. The supernatant was then removed and kept at 4°C. AchE activity was measured by determining the hydrolysis rate of 40 μL supernatant, using 40 μL of acetylthiocholine iodide 2.5 mM (Sigma-Aldrich, USA) and 140 μL phosphate buffer 0.1 M, and 20 μL of the 5, 5′-Dithio-bis-(2-nitrobenzoesAure) (DTNB, Sigma-Aldrich, USA) dissolved in 10 mL Tris-HCl buffer and 15.2 mg of NaHCO_3_. The final volume was 240 μL. The addition of acethylthiocholine initiated the reaction. A microplate reader at 415 nm was used to detect hydrolysis of DTNB, over 5 min (in intervals of 1 min). To determine the protein concentration, the Bradford method was used with bovine plasma albumin (Sigma-Aldrich, USA) [55].

### 2.10. Statistical analysis

The normality of the data was examined using the Kolmogorov-Smirnov test. The data were analyzed by applying one-way analysis of variance (ANOVA) within the SPSS software (ver. 19.0) to compare group means. Two-way ANOVA was applied to identify significant differences in the interactive effects of treatments and time. Furthermore, Duncan’s multiple range tests were carried out. To perform principal component analysis (PCA) between the studied parameters and different treatments in diazinon stress, the PCA test was used in R studio (ver. 1. 3. 1093). A statistical significance was determined using a significance level of 0.05. GraphPad Prism 8 was used to create the graphs. All data were stated as mean ± standard deviation.

## 3. Results

### 3.1. HSP70 gene expression

**Fig 1:**
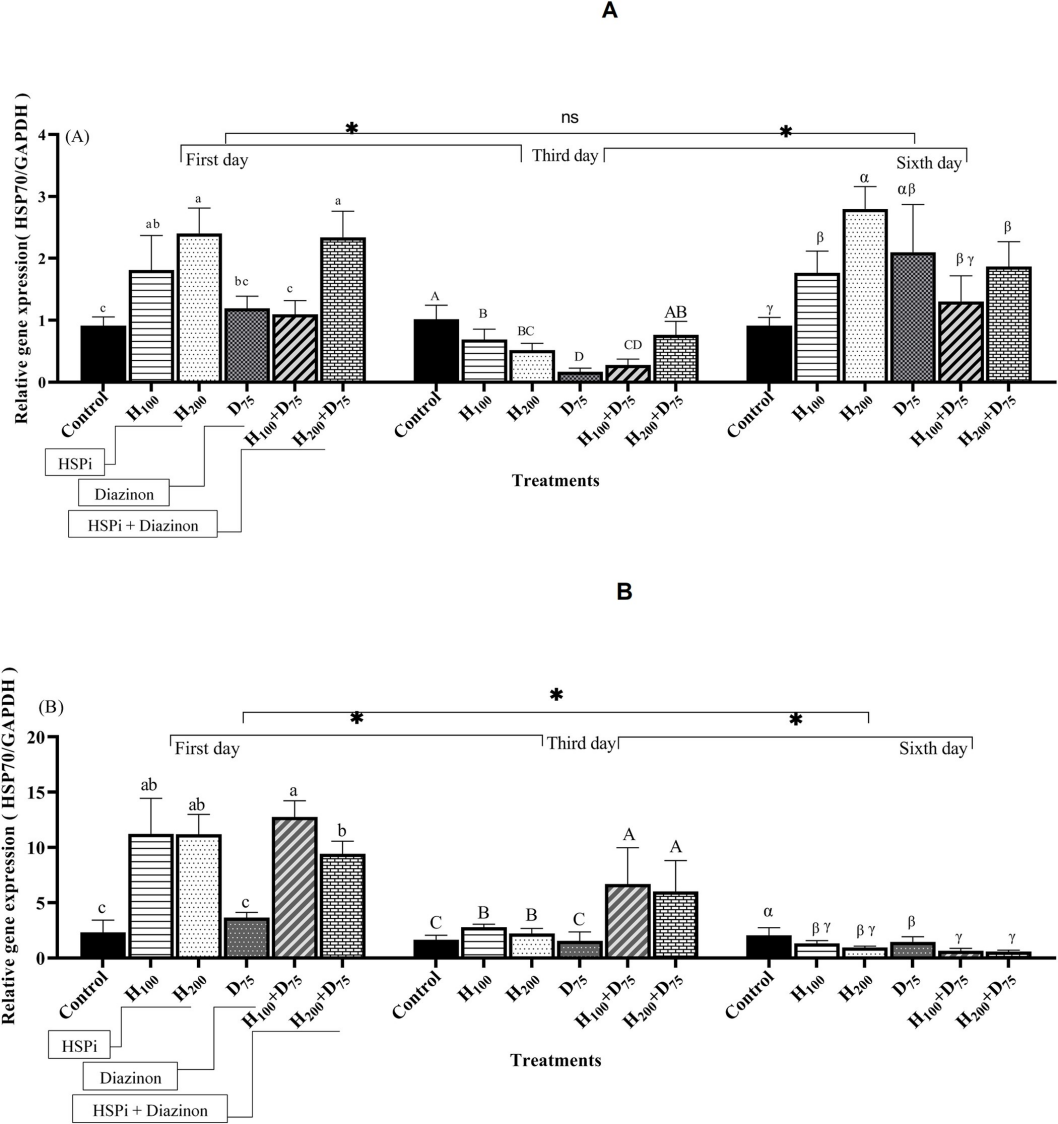
Induction of HSP70 gene expression in the liver (A) and gill (B) of *Acipenser stellatus* exposed to sublethal concentration of diazinon (75% of LC_50_) for six days; Control, fish that were not exposed to any HSPi or diazinon stress; H_100_ and H_200_, fish that received 100 and 200 mg.L^-1^ of HSPi for four hours; D_75_, fish that received 75% of the concentration of LC_50_ diazinon; H_100_+D_75_ and H_200_+D_75,_ samples pre-treated with HSPi compound for 4 hours and then subjected to diazinon. Standard error bars are based on three biological replicates. Different letters are used to indicate the statistical differences between treatment groups. The significant stars denote the mean time comparisons.

In the liver, on the first day after pre-treatment with 100 and 200 mg.L^-1^ HSPi, the expression of the HSP70 gene exhibited a statistically significant rise in comparison to the control group and D75 groups (P < 0.05) (Fig 1A). Furthermore, in fish pre-treated with HSPi and then exposed to diazinon, the HSP70 gene expression under 200 mg.L^-1^ of HSPi, demonstrated a significant increase in comparison to the control, D_75_, and H_100_+D_75_ treatments (P < 0.05) (Fig 1A).

On the third day, there was a noteworthy decrease observed in the expression of the HSP70 gene across all experimental groups in comparison to the control group (P < 0.05). Meanwhile, on the sixth day, the HSP70 gene expression level was remarkably more than that of the control (P < 0.05). However, the expression of gene did not show any noticeable disparity among the H_100_, D_75_, H_100_+D_75_, and H_200_+D_75_ treatments (P > 0.05). The highest level of expression was observed in the H_200_ treatment on the sixth day (Fig 1A).

The two-way ANOVA indicated that there was a noteworthy interaction effect between the treatment and time (Table 2). There was no significant time difference in the average time pairs between days 1 and 6 (P > 0.05). Nevertheless, a significant difference was observed between days 1 with 3 and days 3 with 6 (P < 0.05) (Fig 1A).

**Table 2:** Two-way ANOVA on the effect of treatment and time and their interactions on studied parameters.

	Treatment		Time		Treatment*Time	
	F	p	F	p	F	p
**Liver HSP70**	12.40	<0.05	71.45	<0.05	7.51	<0.05
**Gill HSP70**	3.63	>0.05	176.37	<0.05	14.66	<0.05
**SOD**	1389.43	<0.05	194.63	<0.05	37.86	<0.05
**CAT**	453.90	<0.05	110.84	<0.05	33.69	<0.05
**T-AOC**	162.63	<0.05	35.47	<0.05	20.44	<0.05
**Lysozyme**	433.59	<0.05	308.15	<0.05	40.84	<0.05
**IgM**	200.45	<0.05	143.80	<0.05	15.32	<0.05
**C3**	268.05	<0.05	258.98	<0.05	25.78	<0.05
**Cortisol**	94.28	<0.05	12.71	<0.05	6.54	<0.05
**AChE**	25.51	<0.05	18.15	<0.05	0.47	>0.05

There was a notable rise in the expression of the HSP70 gene in the gill of sturgeon fry that were pre-treated with HSPi, particularly on the first and sixth days of sampling. This increase was significant when compared to both the control group and the D_75_ treatment group (P < 0.05) (Fig 1B). On the first day, fish pre-treated and subjected to diazinon had significantly higher gene expression than control and D_75_. The maximum expression of HSP70 was recorded in fish receiving HSPi and H_100_+D_75_ on the first day (P < 0.05) (Fig 1B).

On the third day after the stress, HSP70 gene expression decreased in all groups. However, it was significantly higher in H_100_+D_75_, and H_200_+D_75_ treatments compared to the control and D_75_ groups (Fig 1B). On the sixth day, HSP70 gene expression significantly reduced after pre-treatment with HSPi and diazinon exposure than the control and D_75_ (P < 0.05) (Fig 1B).

The findings of two-way ANOVA revealed that the main effect of time on HSP70 gene expression was significant and their interaction effects were also significant (P < 0.05) (Table 2).

### 3.2. Activity of antioxidant enzymes

**Fig 2:**
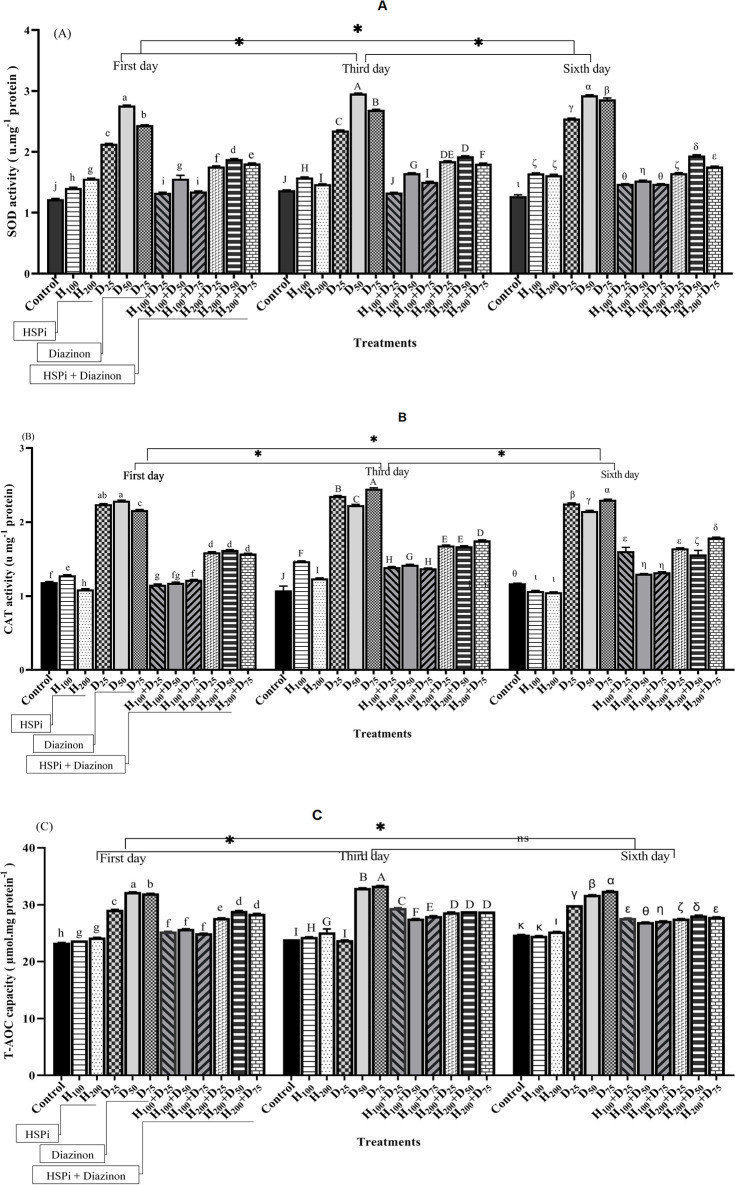
Antioxidant activity, (A) SOD, (B) CAT, and (C) T-AOC, exposed to diazinon for six days in *Acipenser stellatus* fry. Control, fish that were not exposed to any HSPi or diazinon stress; H_100_ and H_200_, fish that received 100 and 200 mg.L^-1^ of HSPi for four hours; D_25_, D_50_, and D_75_ groups, fish that received 25%, 50%, and 75% of the concentration of LC_50_ diazinon; H_100_+D and H_200_+D, samples were pre-treated with HSPi for 4 hours and then subjected to diazinon. Standard error bars are based on three biological replicates. Different letters are used to indicate the statistical differences between treatment groups. Significant asterisks are related to time averages comparison.

During the entire experimental period, fish that were only exposed to diazinon (i.e., D_25_, D_50_, and D_75_) showed the highest SOD activity (P < 0.05) (Fig 2A). In fish pre-treated with HSPi and subjected to diazinon, SOD activity decreased significantly on days 1, 3, and 6. In addition, SOD activity in fish pre-treatment with 100 mg.L^-1^ of HSPi was significantly lower than in fish pre-treatment with 200 mg.L^-1^ (P < 0.05) (Fig 2A). The comparison between fish pre-treated with HSPi and control groups indicated that SOD activity in pre-treated fish increased significantly for three days of sampling. The highest and lowest levels of SOD activity were observed in D_50_ and control groups, respectively.

As shown in Fig 2B, CAT activity in the liver increased in all groups one day after applying diazinon. The highest activity of CAT was recorded in fish exposed to different diazinon concentrations (D_25_, D_50_, and D_75_) for all time points (P < 0.05) (Fig 2B). CAT activity in H_100_ was significantly different from the controls and slightly increased from day 1 to day 3. However, a significant reduction was observed in H_100_ treatment on day 6 (P < 0.05). For H_200_, compared to the control group, CAT activity was significantly lower on the first and sixth days (P > 0.05). But it significantly increased on the third day (P < 0.05) (Fig 2B). The high activity of CAT was observed on the third day in the D_75_ treatment and the lowest level was observed in the control group.

For total antioxidant (T-AOC) on the first day, a lower level was observed in control, H_100,_ and H_200_ groups compared to diazinon-only treatments (P < 0.05) (Fig 2C). The highest amount of T-AOC was detected in diazinon treatments (D_25_, D_50_, and D_75_) during the experimental period (P < 0.05) (Fig 2C). The same T-AOC trend was repeated on the third and sixth days of the experiment. Comparison between the HSPi pre-treatments and control demonstrated a significant difference in T-AOC activity on all three days of sampling (P < 0.05). In fish that underwent pre-treatment with a concentration of 100 mg L^-1^ of HSPi, the activity of T-AOC was found to be significantly decreased compared to that of the group treated with a concentration of 200 mg.L^-1^ (P < 0.05) (Fig 2C).

In addition, the two-way ANOVA showed that the interaction effects of time and treatment had a significant effect on SOD, CAT, and T-AOC activity level (Table 2).

### 3.3. Immune parameters

**Fig 3:**
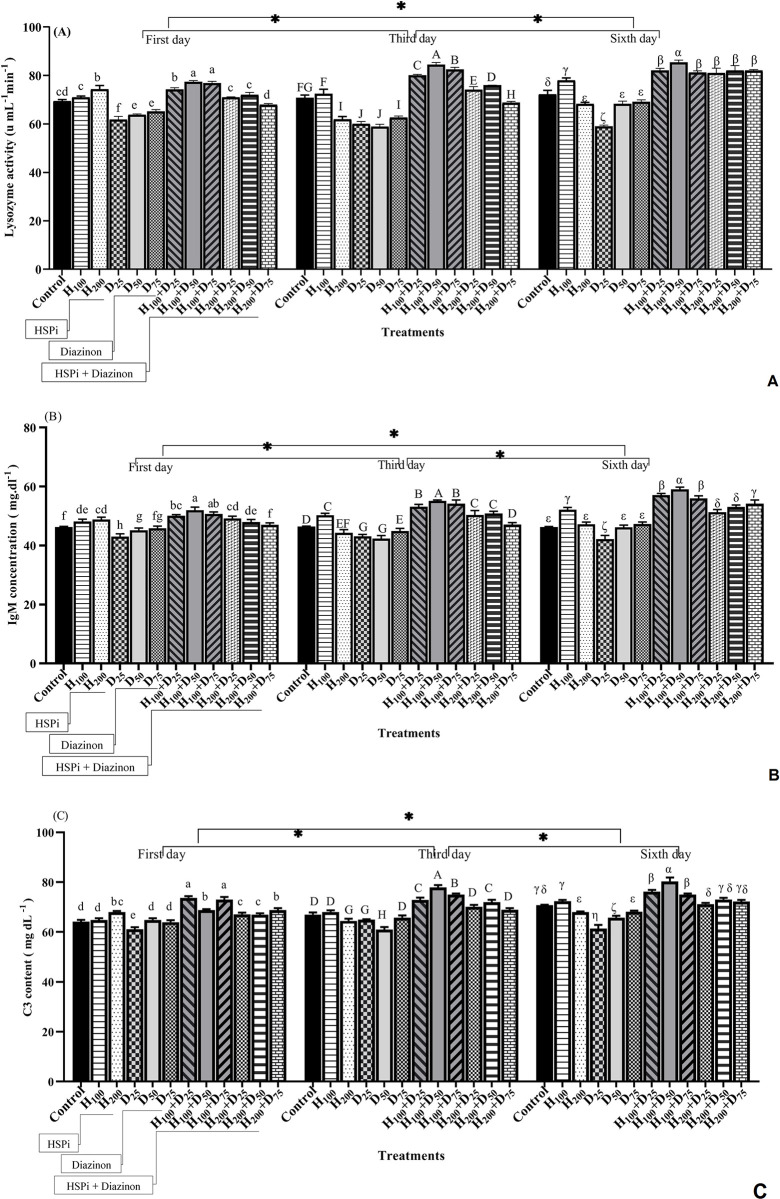
Immune parameters activity (A) lysozyme, (B) IgM, and (C) complement C3, in the serum of *Acipenser stellatus* fry exposed to sublethal concentrations of diazinon (25%, 50%, and 75% of LC_50_) for six days. Control, fish that were not exposed to any HSPi or diazinon stress; H_100_ and H_200_ fish that received 100 and 200 mg.L^-1^ of HSPi for four hours; D_25_, D_50_, and D_75_ groups fish that received 25%, 50%, and 75% of the concentration of LC_50_ diazinon; H_100_+D and H_200_+D samples were pre-treated with HSPi for 4 hours and then subjected to diazinon. Standard error bars are based on three biological replicates. Different letters are used to indicate the statistical differences between treatment groups.

On the first day, lysozyme activity in the HSPi+diazinon groups significantly increased compared to the control and diazinon-only treatments (P < 0.05). Thus, the highest lysozyme activity was observed in the H_100_+diazinon and H_200_+diazinon groups (Fig 3A). On the third and sixth days, the activity of lysozyme in the fish receiving HSPi and then diazinon exhibited a significant increase when compared to the other treatments (P < 0.05). In comparison, the lowest lysozyme activity was observed in diazinon-only treatments (D_25_, D_50_, and D_75_). On the first and third days of sampling, the lysozyme activity in H_100_+diazinon treatments was higher than in the H_200_+diazinon group (Fig 3A).

The pattern of IgM and C3 in the serum of *Acipenser stellatus* during the experiment was similar (Fig 3B and 3C). The IgM and C3 concentrations showed a significant increase in stellate fry receiving HSPi and HSPi+diazinon treatments during the entire experimental period. Here, H_100_+D treatments had the highest amount of IgM and C3 (P < 0.05) (Fig 3B). Fish exposed to diazinon after HSPi pre-treatment showed a significant increase in IgM and C3 concentrations on days 1, 3, and 6 (P < 0.05). The lowest IgM and C3 levels were observed in the fish receiving diazinon. Furthermore, it was observed that the H_200_ group exhibited a significant decrease in both IgM and C3 levels compared to the H_100_ group (P < 0.05) (Fig 3B).

According to the two-way ANOVA, there was a statistically significant interaction between the variables of time and treatment in relation to the levels of lysozyme, IgM, and C3 activity (P < 0.05) (Table 2). There was a notable discrepancy between the means of the time pairs and treatment pairs, as determined by Duncan’s test.

### 3.4. Cortisol

**Fig 4:**
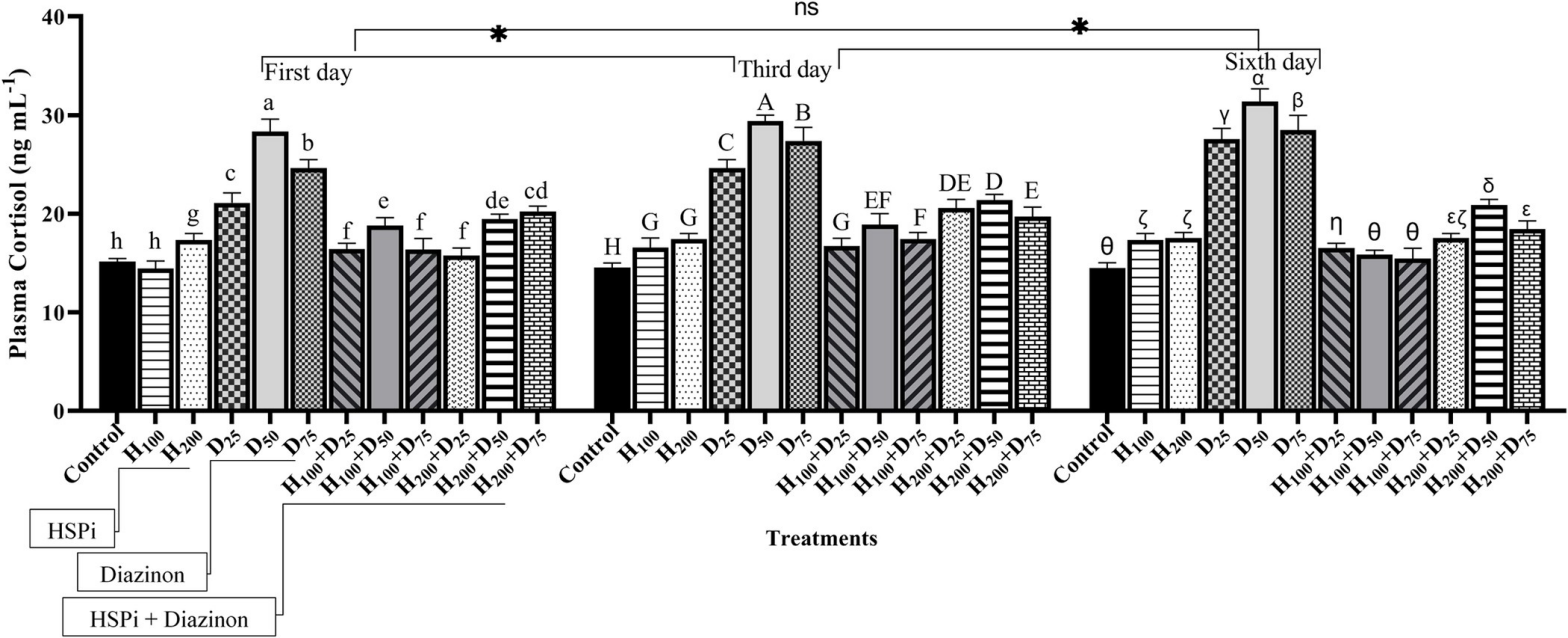
Cortisol levels of stellate sturgeon fry exposed for 6 days to different diazinon concentrations. Control, fish that were not exposed to any HSPi or diazinon stress; H_100_ and H_200_ fish that received 100 and 200 mg.L^-1^ of HSPi for four hours; D_25_, D_50_, and D_75_ groups, fish that received 25%, 50%, and 75% of the concentration of LC_50_ diazinon; H_100_+D and H_200_+D samples pretreated with HSPi for 4 hours and then exposed to diazinon. Standard error bars are based on three biological replicates. Different letters are used to indicate the statistical differences between treatment groups. Significant asterisks are related to time averages comparison.

The highest amount of cortisol was observed in diazinon treatments (D_25_, D_50_, and D_75_) throughout the experiment (P < 0.05) (Fig 4). The level of cortisol in the fish subjected to diazinon after pre-treatment with HSPi was significantly decreased (P < 0.05) (Fig 4). Comparing the control and HSPi pre-treatments indicated that H_200_ remarkably increased the serum cortisol (P < 0.05), although, not as much as diazinon. Based on the two-way ANOVA analysis, there was a considerable interaction observed between time and treatment on cortisol level (P < 0.05) (Table 2). Average cortisol levels were significantly different between all treatment pairs. However, for time pairs, on day 3, cortisol levels were different from day 1 and day 6.

### 3.5. Acetylcholinesterase (AchE)

**Fig 5:**
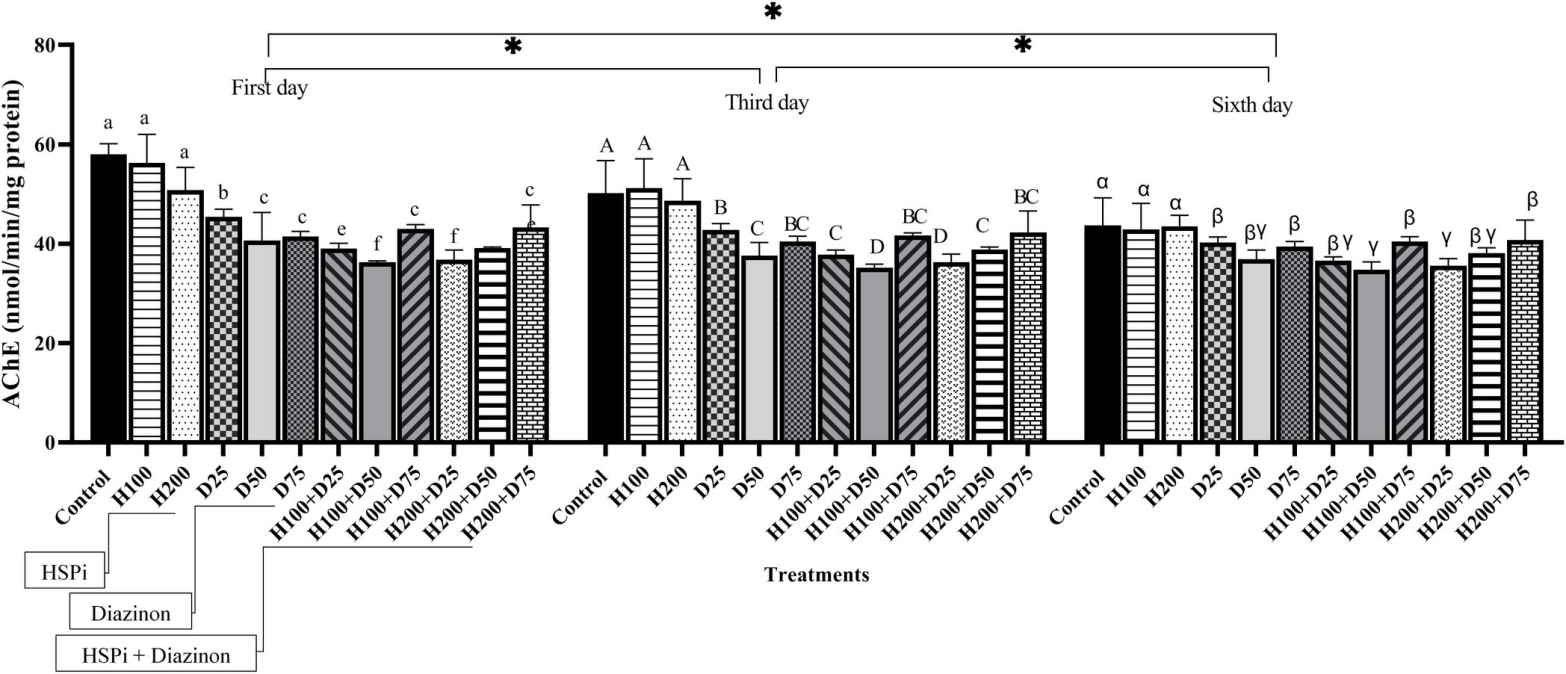
AChE activity in *Acipenser stellatus* fry exposed to different concentrations of diazinon for six days. Control, fish that were not exposed to any HSPi or diazinon stress; H_100_ and H_200_, fish that received 100 and 200 mg.L^-1^ of HSPi for four hours; D_25_, D_50_ and D_75_ groups, fish that received 25%, 50% and 75% of the concentration of LC_50_ diazinon; H_100_+D and H_200_+D samples were pre-treated with HSPi for 4 hours and then subjected to diazinon. Standard error bars are based on three biological replicates. Different letters are used to indicate the statistical differences between treatment groups. The significant stars are related to the comparison of time averages.

Throughout the experiment period, the activity of AchE in the brain showed a significant increase in the control and the fish received HSPi compared to other treated groups (P < 0.05) (Fig 5). The alterations in AchE followed a consistent pattern across various treatments and on different days (1^st^, 3^rd^, and 6^th^) (Fig 5). However, there was no significant difference among fish receiving diazinon and HSPi+diazinon. The outcomes of the two-way ANOVA revealed no substantial interaction between time and treatment on the activity of AchE (P > 0.05), whereas treatment and time separately had a significant effect (P < 0.05) (Table 2).

### 3.6. Principal component analysis (PCA)

**Fig 6:**
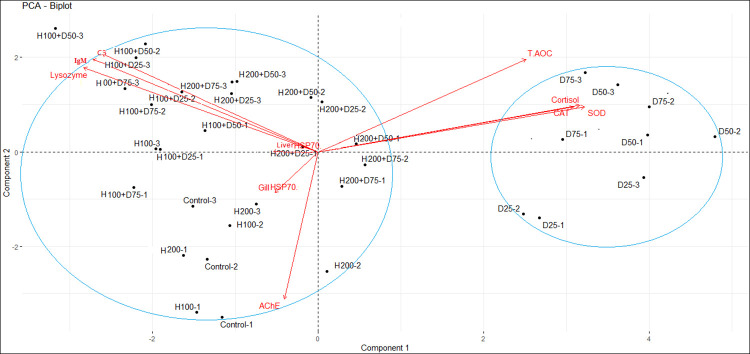
Biplot graph resulting from principal component analysis (PCA) for diazinon stress. 1, 2, and 3 represent sampling times.

The principal component analysis was performed based on 10 variables and 36 treatments. The first and second principal components account for 49.25% and 23.68% of the variation, respectively (Table 3). Considering a score higher than 0.7, lysozyme, IgM, C3, SOD, CAT, T-AOC, and cortisol variables were important factors in determining the relationship between treatments and tested variables in the first component (Table 4). The results of this analysis were separated into two main groups (Fig 6). The first group consisted of treatments D_25_, D_50_, and D_75_, while the second group included the remaining treatments. The first group showed the highest correlation with cortisol, CAT, SOD, and T-AOC variables, while the HSPi+diazinon treated groups had the greatest impact on immune parameters (Fig 6).

**Table 3:** Eigenvalue, percentage of variance and cumulative percentage of variance related to all three components.

	Component 1	Component 2	Component 3
Eigenvalue	4.92	2.36	1.14
Percentage of Variance	49.25	23.68	11.47
Cumulative Percentage of Variance	49.25	72.93	84.41

**Table 4:** Scores of each variable in the main components.

Variable	Score in Component 1	Score in Component 2
Lysozyme	-0.81	0.51
IgM	-0.78	0.56
C3	-0.75	0.59
SOD	0.93	0.27
CAT	0.89	0.27
T-AOC	0.72	0.56
Cortisol	0.91	0.28
Liver HSP70	-0.11	-0.90
HSP70 Gill	-0.05	0.01

## 4. Discussion

This study aimed to investigate the protective effects of a heat shock protein (HSP) inducer (HSPi) on stellate sturgeon (*Acipenser stellatus*) fry against diazinon-induced stress. The HSP70 family is the most extensively studied HSP in aquatic organisms [56]. HSP70 plays a crucial role in protecting cellular components from damage caused by stress, as it effectively prevents protein aggregation while also facilitating proper protein folding [57]. TEX-OE^®^ is a patented prickly pear cactus extract (*Opuntia ficus indica*) and an alternative method to enhance HSP70 in the body through HSP induction [58]. Several studies have shown that TEX-OE^®^ accelerates HSP70 synthesis in fish [3,5] and shellfish [8,58,59], enhancing their resistance to various stressors. In a different study, the effect of HSPi on HSP70 modulation was analyzed in *Artemia franciscana nauplii*, which showed that HSPi induced HSP70 expression both in a concentration and time-dependent approach [60]. Another evaluation of the Tex-OE^®^ compound on *Artemia franciscana’s* interaction with pathogenic *Vibrios* demonstrated increased protection against *Vibrio campbellii*, although long-term exposure decreased their survival. Likewise, in a study evaluating the effect of HSPi (TEX-OE^®^) on the HSP70 gene expression in the gill, intestine, and liver of *Acipenser persicus* during the 7-day post-injection period with the bacteria *Aeromonas hydrophila*, HSPi significantly increased HSP70 expression in a dose-dependent manner [9]. In another study, Tex-OE^®^ rapidly increased the levels of HSPs in fingerlings of common carp before acute ammonia stress [58]. In the present study, 4 hours of pre-exposure to Tex-OE^®^ increased HSP70 gene expression in both liver and gills of stellate sturgeon fry, especially on the first day. The upregulation of HSP70 expression in fish subjected to diazinon suggests that the pesticide acts as a stressor, triggering the cellular stress response pathway. This upregulation is believed to be a protective mechanism to mitigate the potential damage caused by diazinon-induced stress. Furthermore, the effectiveness of Tex-OE^®^ in increasing HSP70 gene expression in liver tissues was higher than that in the gills on the sixth day, this may be attributed to the direct exposure of the gills to the surrounding environment, leading to a rapid rise in expression of the HSP70 on the first day. It is significant to note that persistent stress might lessen the capacity of the stress response, which includes the generation of HSP70. In cases of chronic stress, even an increased level of HSP70 may not be enough to protect fish from the detrimental consequences of stress, thereby making them more prone to stress-related complications.

Oxidative stress can occur when there is an imbalance between the generation of reactive oxygen species (ROS) and the quantities of antioxidants, ultimately leading to cellular harm [60,61]. According to a study by [62], crucian carp exposure to 0.3 mg.L^-1^ of diazinon per day for 21 days induced oxidative stress. In one experiment, larvae of zebrafish subjected to diazinon concentrations reaching from 0.002 to 0.02 mg.L^-1^ showed differences in the activity of oxidative enzymes and gene expression, with no detectable CAT activity and significantly increased expression of oxidative enzymes [26,63]. Another study evaluated the effects of sublethal doses of diazinon (0, 0.8, 1.6, and 3.2 mg.L^-1^) on zebrafish after 30 days of treatment, and found significant increases in the expression of immune and antioxidant (SOD and CAT) genes [18]. In this study, we found that the fish treated with all three sublethal concentrations of diazinon had higher antioxidant enzyme activity, but this activity decreased when the fish were first treated with HSPi and then exposed to diazinon. This suggests that the increase in HSP70 due to Tex-OE^®^ treatment may play an important role in controlling ROS production and regulating the activity of antioxidant enzymes. Similarly, in *Labio rohita* fingerlings subjected to starvation stress, HSP70 gene expression and antioxidant enzyme activity increased. This implies that HSP70 could aid in preserving the stability of cells by reducing the impact of oxidative stress caused by starvation [64].

The immune response in fish can be deregulated by pollutants like diazinon existing in aquatic ecosystems [35,65,66]. Sharifpour et al., [67] found that the decreased immunity observed in the fish that were exposed to diazinon confirmed the immunosuppressive effects of this substance. In the current study, the immunity status, such as lysozyme, IgM, and C3 was reduced in the diazinon groups (D_25_, D_50_, and D_75_) compared to control. It is believed that these immunosuppressive effects occur primarily by inhibiting the synthesis of key components of the immune system [19,66,68]. Diazinon has a suppressive effect on fish cellular immunity, which is achieved by reducing lymphocyte proliferation. The decrease in lymphocytes may be due to the release of cortisol in reaction to stress, which causes lymphocyte apoptosis, or their redistribution from the blood to the tissues [69]. The reported reduction in IgM levels in response to diazinon may be due to diazinon inhibitory effects on lymphocyte proliferation, which is responsible for IgM production in hematopoietic tissues [70]. On the first, third, and sixth days of the experiment, it was observed that the H100+D groups showed the most elevated levels of lysozyme, IgM, and C3. Recent studies have shown that HSPs stimulators or inducers have a significant impact on both innate and acquired immunity, and possess immuno-enhancing properties [8,13]. Notably, HSP70 plays a critical role in the immune function of fish. Studies have reported that HSP70 can stimulate white blood cells and antibodies, leading to improved infection-fighting abilities. The active expulsion of HSP70 to the outside of the cells or the induction of HSP70 can activate both the innate and adaptive immune systems [71]. Additionally, HSPs have been shown to generate pro-oxidant activity and induce protective immune responses in crustaceans [8]. Fish with higher HSP70 expression levels showed enhanced activity of immune parameters such as serum complement and immunoglobulin levels, as well as antioxidant enzyme activities [72]. Studies have also demonstrated that the concentration of IgM, complement C3, and lysozyme can rise after exposure to HSP inducer in Persian sturgeon fry [5]. Moreover, the innate immune system genes of prophenoloxidase and transglutaminase can be up-regulated in species like *Artemia franciscana* when responding to HSP70 induction [60]. HSP70 expression was found to interact with other immune parameters and antioxidant enzymes, improving the fish’s ability to adapt to the environment [73]. It seems that in fish, HSP70 has the potential to improve the functioning of the immune system through triggering the release of cytokines via signal transduction when recognized by immune cells [13]. However, the extent of the benefit relies on different factors, including chronic exposure to stress, which can impair the immune response and reduce the amount of HSP70 produced in the organism. Therefore, if stress persists, the protective effect of high levels of HSP70 might not sufficiently protect the fish from damage caused by stress, leaving it vulnerable to illnesses and other complications associated with stress.

To respond efficiently to stressors, cortisol is the most common corticosteroid generated by the hypothalamus-pituitary-adrenal/internal (HPI) axis in humans and fish [74]. In our study, the significant reduction of cortisol levels in the HSPi+diazinon groups supports the anti-stress role of HSPi in dealing with diazinon stress, resulting in a return of cortisol levels to normal condition in fish that received HSPi before diazinon stress. Cortisol may lead to changes in HSP70 levels in fish tissues during periods of physiological stress, suggesting a correlation between the stress reactions in the nervous and endocrine systems with those at the cellular level [10]. The synthesis of HSPs is controlled by an intricate system of communication pathways, such as the HPI axis, which regulates the release of cortisol [75]. When the functioning of the HPI axis decreases, it results in a cycle of negative feedback that impacts the production of cortisol [76]. There is evidence that suggests HSP70 and the glucocorticoid receptor (GR) connect and produce a stable HSP70-GR-HSP90 molecular structure in the cytosol [75,77], which finally inhibits cortisol production. Our research indicates that HSPs and cortisol interact as chaperone and client, respectively, meaning that HSPs help to modulate the activity of cortisol during periods of stress. The fish that were treated with HSPi+diazinon exhibited less cortisol activity compared to diazinon (D_25_, D_50_ and D_75_) treated groups.

Measuring AChE activity in fish brains is a method of diagnosing pesticide exposure, particularly organophosphates that inhibit anticholinesterase enzymes in aquatic environments [21,65]. The inhibition of AChE activity suggests that these pollutants can interfere with crucial processes such as nerve cell energy metabolism. Furthermore, diazinon inhibition of AChE can affect other metabolic activities in animals, resulting in death [78]. Evaluating HSP70 and AChE in a molluscan species (*Geukensia demissa*) over time has revealed a relationship between chaperone and client proteins of the HSP70/AChE system, demonstrating various adaptive mechanisms used by organisms to tolerate habitats subjected to environmental change [79]. Some researchers have linked organophosphates such as diazinon to changes in neurotransmission in fish [66,80,81]. Chronic exposure to a non-lethal concentration of diazinon significantly inhibits AChE activity in the brain of *Oreochromis niloticus* [82]. In *Oreochromis niloticus*, acute exposure to diazinon significantly decreases AChE activity [83]. According to the current study, diazinon significantly decreases AChE levels in stellate sturgeon fry brain. Pre-exposure to HSPi did not restore enzyme activity to normal levels. Overall, it appears that HSPi at concentrations of 100 and 200 mg.L^-1^ are insufficient to affect AChE activity or moderate the neurotoxic effects of diazinon. However, a higher concentration of HSPi may increase AChE activity in fish under diazinon-induced stress.

## 5. Conclusion

The findings of this study indicate that prior exposure to Tex-OE^®^ can result in the upregulation of the HSP70 gene in stellate sturgeon fry. This increase in expression offers improved protection against pesticide exposure and boosts their biochemical and immunological defense mechanisms. The application of an HSP inducer, such as Tex-OE®, can potentially enhance the overall health of valuable sturgeon and aid in their ability to cope with environmental stressors like diazinon, a pesticide. Given the high mortality rates observed when sturgeon fry are released into the river from artificial breeding centers and exposed to environmental stressors, it is recommended to expose them to HSP-inducing compounds before releasing them into the natural environment. This pre-exposure can offer optimal conditions for their survival and improve their chances of adapting to the challenges they may face in the wild.
